# Predicting bladder cancer survival with high accuracy: insights from MAPK pathway-related genes

**DOI:** 10.1038/s41598-024-61302-0

**Published:** 2024-05-07

**Authors:** Guangyang Cheng, Zhaokai Zhou, Shiqi Li, Shuai Yang, Yan Wang, Zhuo Ye, Chuanchuan Ren

**Affiliations:** Department of Urology, The First Affiliated Hospital of Zhengzhou University, Zhengzhou, 450052 Henan China

**Keywords:** Prognostic markers, Bioinformatics

## Abstract

The mitogen-activated protein kinase (MAPK) pathway plays a critical role in tumor development and immunotherapy. Nevertheless, additional research is necessary to comprehend the relationship between the MAPK pathway and the prognosis of bladder cancer (BLCA), as well as its influence on the tumor immune microenvironment. To create prognostic models, we screened ten genes associated with the MAPK pathway using COX and least absolute shrinkage and selection operator (LASSO) regression analysis. These models were validated in the Genomic Data Commons (GEO) cohort and further examined for immune infiltration, somatic mutation, and drug sensitivity characteristics. Finally, the findings were validated using The Human Protein Atlas (HPA) database and through Quantitative Real-time PCR (qRT-PCR). Patients were classified into high-risk and low-risk groups based on the prognosis-related genes of the MAPK pathway. The high-risk group had poorer overall survival than the low-risk group and showed increased immune infiltration compared to the low-risk group. Additionally, the nomograms built using the risk scores and clinical factors exhibited high accuracy in predicting the survival of BLCA patients. The prognostic profiling of MAPK pathway-associated genes represents a potent clinical prediction tool, serving as the foundation for precise clinical treatment of BLCA.

## Introduction

BLCA is one of the most frequently occurring cancers and is the most dominant malignant tumor in the urinary system, ranking in the top ten^1^. Every year, there are about 550,000 fresh instances of BLCA documented worldwide, making up roughly 3.0% of new cancer detections and 2.1% of fatalities caused by cancer^2^. The majority of newly diagnosed cases (75%) are non-muscle-invasive (NMIBC), while 25% are muscle-invasive (MIBC)^3^. NMIBC has a relatively better prognosis but tends to recur frequently^4^. Growing studies indicate that individuals diagnosed with MIBC encounter a worse outlook and a heightened likelihood of metastasis, resulting in a survival rate below 50% within five years^5–7^. Despite established treatment options, radical cystectomy, and pelvic lymph node dissection, approximately 50% of patients who undergo surgery for MIBC often face recurrence afterward, primarily caused by distant metastases^8^. Hence, early disease diagnosis and the identification of prognostic markers are crucial for managing BLCA effectively. In recent years, various signaling pathway-related models like the Notch pathway, EMT pathway, TGF-β pathway, and PI3K pathway have been established to predict the survival of BLCA patients^9–12^. However, a risk profile related to the MAPK pathway for predicting survival in BLCA patients has not yet been established.

In mammalian cells, MAPK signaling is a fundamental mechanism, transmitting signals related to proliferation, apoptosis, and differentiation^13–15^. ERK, p38, JNK, and ERK5, which are a set of serine-threonine kinases conserved throughout evolution, serve as the classical MAPK pathways^16^. These pathways involve different MAPKs associated with specific MAPK kinases (MAPKK) and MAPK-kinase-kinase (MAPKKK), forming a conserved tertiary enzymatic cascade (MAPKKK → MAPKK → MAPK)^17–19^. Aberrant mutations in certain components of the MAPK pathway have been identified as significant contributors to various cancers^20,21^. Consequently, intervention in this pathway has been explored as a strategy for tumor therapy.

Previous research indicates that 45% of potential therapeutic targets in BLCA are related to the MAPK pathway^22^, and this association correlates with the prognosis in BLCA^23^. In this study, a prediction model was developed based on genes associated with the MAPK signaling pathway. Importantly, we aimed to investigate the potential mechanisms by which the MAPK signaling pathway affects prognosis and immunotherapy response. Our findings provide a foundation for future advancements in precision medicine for BLCA.

## Methods

### Data acquisition

From the Genomic Data Commons (GDC) database, we acquired RNA-seq data and clinical information for 403 tumor tissue samples and 19 normal tissue samples. A log2 (TPM + 1) transformation was applied to the downloaded transcripts per million (TPM) data, and genes with total expression values less than 1 in all samples were excluded. The GEO database was used to retrieve expression profiles and clinical data for GSE32894, GSE32548, and GSE48075. We combined these three GEO cohorts into one meta-cohort for subsequent analysis. Probe IDs were converted to corresponding gene symbols, and batch effects were mitigated using the “sva” R package. For clinical data, patients with a survival time of fewer than 30 days and those with missing essential information were excluded, as detailed in Supplementary Table S1. Data for the IMvigor210 cohort is obtained through the IMvigor210CoreBiologies R package. MAPK pathway-related genes were obtained from the Kyoto Encyclopedia of Genes and Genomes (KEGG) database (Supplementary Table S2). The study workflow is illustrated in Supplementary Fig. S1A.

### Differential expression analysis

The analysis of differential expression in TCGA-Counts data was conducted using the “DESeq2” R package. Differential genes meeting the criteria (|log2FC|> 1 and adj.*p* < 0.05) were identified and visualized in a volcano plot. To obtain the intersection between these differentially expressed genes (DEGs) and MAPK-related genes, the “VennDiagram” R package was employed to generate a Venn plot.

### Construction and validation of prognostic gene signatures

The TCGA dataset was divided into training and testing sets randomly, with a ratio of 7:3. The intersected genes underwent uni-variate Cox regression analyses using the “Survival” R package. A LASSO regression analysis was performed using the “glmnet” R package to determine the final model genes and calculate correlation coefficients for each gene after screening for genes with prognostic significance. Risk scores were computed for the training group, validation group, TCGA cohort, and GEO-meta cohort by utilizing the formula: $riskscore={\sum }_{i=1}^{n}ki*Xi$, in which k denotes the relative expression level of the model genes, and X signifies the regression coefficients. Afterward, the patients were categorized into groups of high-risk and low-risk, using the median of the risk score from the training group as the threshold value. The distribution of risk scores and a heatmap for all cohorts were plotted to visually present the results.

### Tumor immune infiltration analysis

Application of the CIBERSORT function of the "IOBR "R package to perform immune infiltration analysis, and the ESTIMATE function to calculate the immune score and stromal score^24–26^.

### Construction of nomograms

A nomogram was created by utilizing the 'rms' R package, which included age, pathological stage, and risk score. The total score was calculated based on the contributions of these independent factors in the nomogram, aiming to predict the corresponding survival rate for patients with BLCA. The accuracy of the nomogram predictions was assessed using calibration curves.

### Tumor mutation analysis and immunotherapy analysis

Tumor mutational burden (TMB) quantifies the number of non-synonymous mutations in somatic cells within a specific genomic region, indirectly reflecting a tumor's capacity and extent for neoantigen production. TMB serves as a predictive indicator for the effectiveness of immunotherapy across a broad spectrum of tumors^27,28^. Simple nucleotide variant datasets from BLCA patients were obtained from the GDC website, and TMBs for individual samples were calculated using the “maftools” R package. Drug sensitivity analysis data were sourced from the Genomics of Drug Sensitivity in Cancer (GDSC) website. The relationship between high and low-risk groups and IC_50_ values of anticancer drugs was analyzed using the "oncoPredict" R package.

### GEPIA website and GSCA website

We employed the Gene Expression Profiling Interactive Analysis (GEPIA) website for mapping the Hazard Ratios (HR) of model genes across various cancers^29^. Additionally, the Gene Set Cancer Analysis (GSCA) website was utilized for conducting analyses on Single Nucleotide Variations (SNV), Copy Number Variations (CNV), immune infiltration, and drug sensitivity related to the model genes^30^.

### Gene set enrichment analysis (GSEA)

The “c2.cp.kegg.v2023.1.Hs.entrez” gene set used for GSEA was downloaded from The Molecular Signatures Database (MSigDB) database and analyzed for differences in different subgroups and then sorted according to log2FoldChange for GSEA analysis, for single gene GSEA groupings were categorized according to the expression median of the gene.

### Human protein atlas database

HPA database stores massive amounts of protein data from human tissues. In this study, we utilized the HPA database to retrieve histopathological data associated with the model genes.

### Clinical sample acquisition

The study received approval from the Ethics Committee of the First Affiliated Hospital of Zhengzhou University, and all volunteers signed informed consent forms before participation. This study adhered strictly to the ethical principles for medical research involving human subjects, as outlined in the Declaration of Helsinki^31^. Clinical samples were sourced from the First Affiliated Hospital of Zhengzhou University, involving patients previously diagnosed with BLCA through pathological examination. Paracancerous tissues were collected from normal tissues within a 3 cm region near the tumor. Following sampling, tissue samples were promptly preserved in liquid nitrogen and transferred to a – 80 °C refrigerator to maintain their integrity for subsequent analyses.

### Quantitative real-time PCR experiments

Total RNA was extracted from the collected BLCA tumor tissues and adjacent normal tissues using the RNAeasy™ Animal RNA Extraction Kit (Beyotime). Subsequently, the reverse transcription process was performed using the PrimeScript™ RT reagent Kit (Takara), and qRT-PCR was conducted with the TB Green® Premix Ex Taq™ II Kit (Takara), following the manufacturer's instructions. The primer sequences are shown in Table 1.

**Table 1:** The list of the primers used for qRT-PCR.

Gene symbol	Forward or reverse primer	Primer sequence (5'–3')
GAPDH	Forward	GGAAGCTTGTCATCAATGGAAATC
	Reverse	TGATGACCCTTTTGGCTCCC
NRTN	Forward	ACCCTGGACGCCCGGATT
	Reverse	CGCAGTAGCGGAACAGCACC
MAP3K8	Forward	TCGCTCAGCCTATCCCTCCTA
	Reverse	GTTCCAGCTCCTTCCTACTCAG
RAC3	Forward	CTCCTACCCCCAAACTGACG
	Reverse	TTCACAGAGCCCACCAATCTC
PDGFD	Forward	GGTGAAAGGAAACGGCTACG
	Reverse	CTCTAATAATGGTACTGGTTTCGGA
JUN	Forward	TGGGTGCCAACTCATGCTAA
	Reverse	TTCTTCGTTGCCCCTCAGC
MAP3K20	Forward	GTTAGATACTCTGAGGATGCGGC
	Reverse	GTTGATACTTAATGGGCACCTGG
IGF1	Forward	GGTGGATGCTCTTCAGTTCGT
	Reverse	GCAATACATCTCCAGCCTCCTTA
PTPRR	Forward	GCAGGAATAGGTAGAACAGGGTG
	Reverse	GCACCATTCCACCTCTATCCA
DUSP2	Forward	TGCTGTCCCGATCTGTGCT
	Reverse	CAGGAACAGGTAGGGCAAGA
PDGFRA	Forward	CTTTGGATTGAACCCTGCTGA
	Reverse	GACATCTCGTGCCAACTCCA

### Statistical analysis

Bioinformatics analysis was performed using R version 4.3.1. The comparison of continuous data utilized either the student’s *t*-test or the Wilcoxon test, depending on the nature of the data, with statistical significance established at a two-sided *p*-value < 0.05.

### Ethical approval

All ethical aspects of this study were approved by the Ethics Committee of the First Affiliated Hospital of Zhengzhou University.

### Consent to participate

Informed consent was obtained from all individual participants included in the study.

## Results

### Differential expression analysis of MAPK pathway-related genes

**Figure 1:**
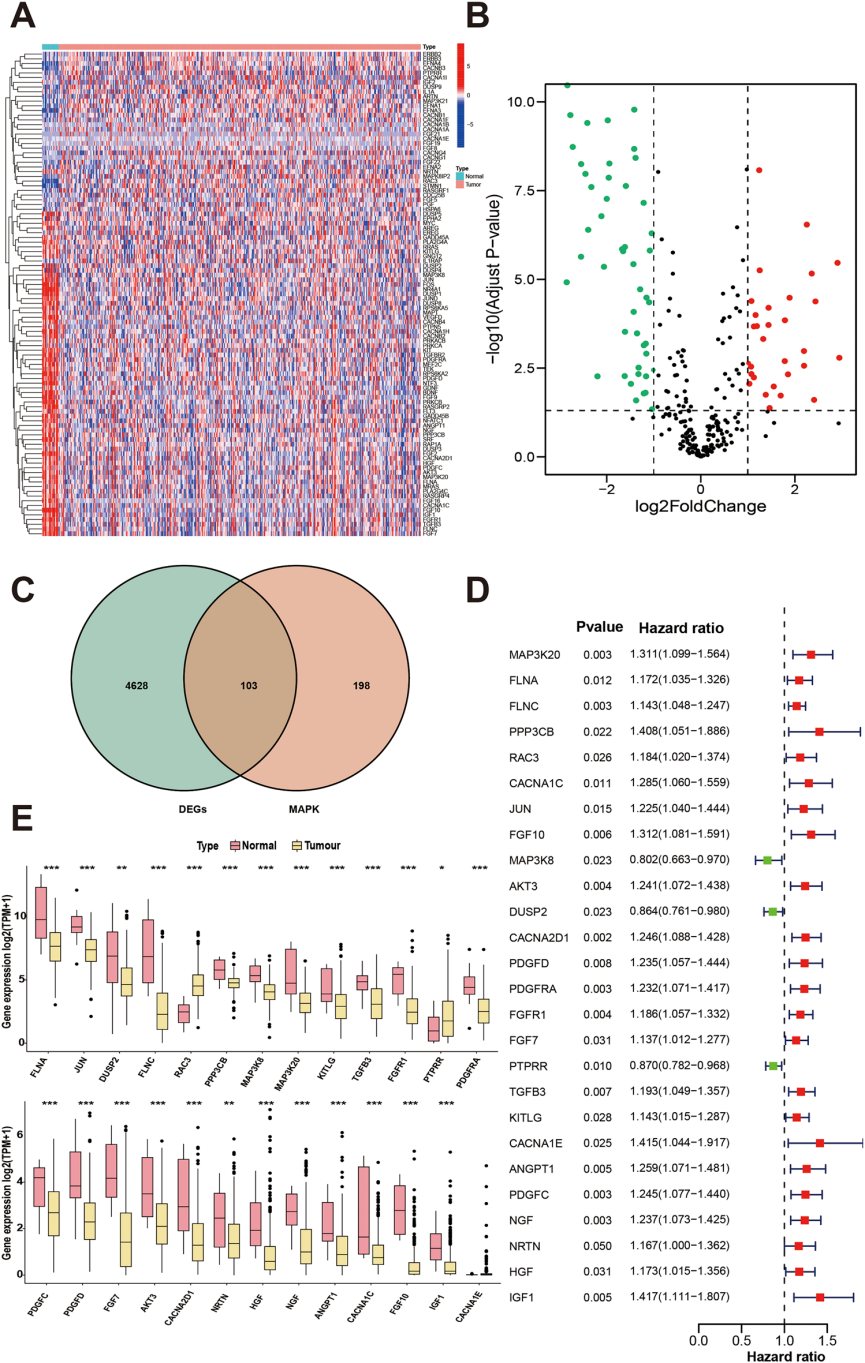
Screening of survival-related genes. (**A**) Heatmap displaying the differential expression of genes associated with the MAPK pathway. (**B**) Volcano plot illustrating the differential expression of MAPK pathway-related genes. (**C**) Venn diagram depicting the intersection of MAPK pathway-related genes with differentially expressed genes. (**D**) Forest plot representing the results of COX regression analysis. (**E**) Box plots showing the expression levels of survival-related MAPK pathway genes in tumor versus normal tissues.

To analyze the DEGs in TCGA-BLCA tumor tissues and normal tissues, RNA-seq data from log-transformed TCGA were subjected to analysis using the "Deseq2" R package. A total of 4731 DEGs were identified, applying criteria of |log2FC|> 1 and adj.*p* < 0.05. By intersecting these DEGs with MAPK-related genes, 103 intersected genes were obtained. The analysis resulted in the identification of these 103 genes, and Venn plots (Fig. 1B), volcano plots (Fig. 1C), and a heatmap (Fig. 1A) illustrating the expression of these 103 differential genes were generated.

### Development and validation of prognostic gene signatures

**Figure 2:**
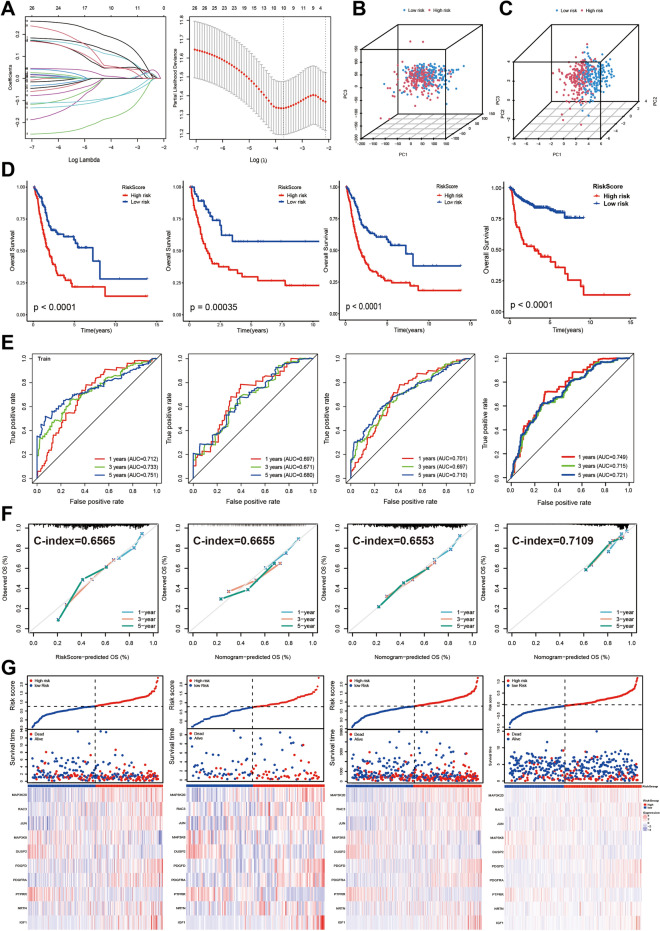
Construction of MAPK prognostic gene signature and survival analysis. (**A**) LASSO regression correlation coefficient and LASSO regression screening model genes for 26 survival-related genes, with the best parameter (lambda min) as the first dashed line on the left. (**B**) PCA analysis of the clustering effect of all genes. (**C**) PCA analysis of the clustering effect of model genes. (**D**) Kaplan–Meier survival analysis of the TCGA-BLCA training cohort, the validation cohort, the overall cohort, and the GEO-meta cohort for high-risk and low-risk groups. (**E**) ROC curves for the TCGA-BLCA training cohort, validation cohort, overall cohort, and GEO-meta cohort. (**F**) Risk score calibration plots for the TCGA-BLCA training cohort, validation cohort, overall cohort, and GEO-meta cohort. (**G**) Risk score distribution plots for the TCGA-BLCA training cohort, validation cohort, overall cohort, and GEO-meta cohort.

During the analysis of the 103 intersecting genes, COX regression analysis was performed, resulting in the identification of 26 genes significantly associated with survival (*P* < 0.05), as depicted in Fig. 1D. The differential expression of these genes between the tumor and normal groups is illustrated in Fig. 1E. Subsequent LASSO regression analysis on the 26 prognosis-related genes revealed 10 candidate genes at the minimum lambda value (Fig. 2A). Risk scores were then calculated for each patient based on the mRNA expression levels of these 10 genes (MAP3K20, RAC3, JUN, MAP3K8, DUSP2, PDGFD, PDGFRA, PTPRR, NRTN, and IGF1) and the corresponding coefficients from the LASSO regression analysis. Using the median value of the risk score from the training cohort as the cutoff, patients across different cohorts were categorized into two groups. Principal Component Analysis (PCA) demonstrated the efficacy of the model genes in clustering patients within the TCGA-BLCA dataset (Fig. 2B,C). The Kaplan–Meier analysis demonstrated a notable decrease in the likelihood of survival in the high-risk group when compared to the low-risk group across these cohorts (Fig. 2D). Receiver operating characteristic (ROC) curves yielded an Area Under the Curve (AUC) value of 0.751 for survival at 5 years (Fig. 2E). The C-index values of RiskScore for the different cohorts are 0.6565, 0.6655, 0.6553, and 0.7109, respectively, and we also plotted the calibration curves, all of which indicate the robustness of the MAPK model (Fig. 2F). The prognostic gene expression profiles are presented in a heatmap (Fig. 2G).

### Association of risk profiles with the tumor microenvironment

**Figure 3:**
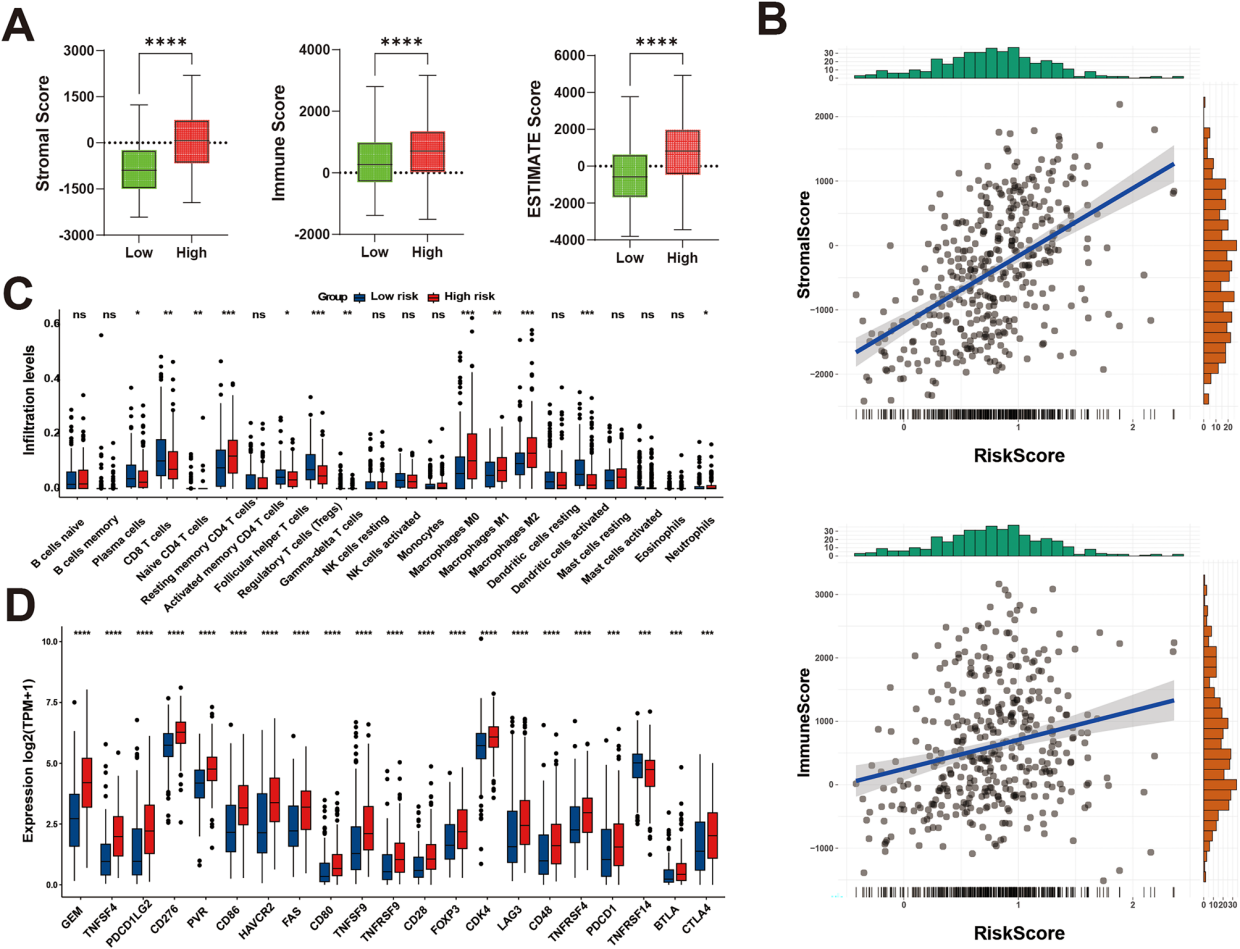
Relationship between risk models and tumor immune microenvironment. (**A**) ESTIMATES analysis of differences in StromalScore, ImmuneScore, and ESTIMATEScore between high and low-risk groups. (**B**) Correlation of risk scores with StromalScore and ImmuneScore. Histograms on the horizontal axis show the distribution of samples with different risk scores, and histograms on the vertical axis show the distribution of samples with different StromalScore and ImmuneScore. (**C**) Box plots of immune cell infiltration in the high-risk group versus the low-risk group (adj.*p* < 0.05). (**D**) Box plots of immune checkpoint expression levels in two groups (adj.*p* < 0.05).

To further investigate the differences in immune infiltration between the two subgroups, the CIBERSORT algorithm was employed to calculate the proportion of immune cell infiltration for all samples, as shown in Supplementary Fig. S1B. Additionally, StromalScore and ImmuneScore were determined for all samples using the ESTIMATE algorithm, and correlation analyses showed higher StromalScore, ImmuneScore, and ESTIMATEScore in the high-risk group (Fig. 3A). Further correlation analysis demonstrated a positive correlation between risk scores and both stromal scores and immune scores, as well as a positive correlation with the ESTIMATEScore (Fig. 3B). This result suggests the presence of a more complex microenvironment in the high-risk group, which may be associated with tumor aggressiveness, treatment resistance, and poorer prognosis. Analysis of the differences in immune infiltration between the two risk groups revealed that patients in the high-risk score group exhibited a higher percentage of resting memory naive B cells, CD4 T cells, M0 macrophages, M1 macrophages, and M2 macrophages. These cell types are associated with an immunosuppressive milieu and may contribute to immune escape from tumors by promoting^32,33^. In contrast, plasma cells, CD8 T cells, regulatory T cells, and activated dendritic cells were more prevalent in patients in the low-risk score group (Fig. 3C), suggesting a more active anti-tumor immune response in the low-risk group. Finally, an analysis of the differences in the expression of 34 immune checkpoints between the high-risk and low-risk groups was conducted (Supplementary Table S3, adj.*p* < 0.05). Among these, 21 immune checkpoints with *P* < 0.01 were selected and plotted in a box line plot. All of them were found to be highly expressed in the high-risk scoring group, except for TNFRSF14 (Fig. 3D), suggesting that the high-risk group may further inhibit effective anti-tumor immune responses.

### Nomogram construction

**Figure 4:**
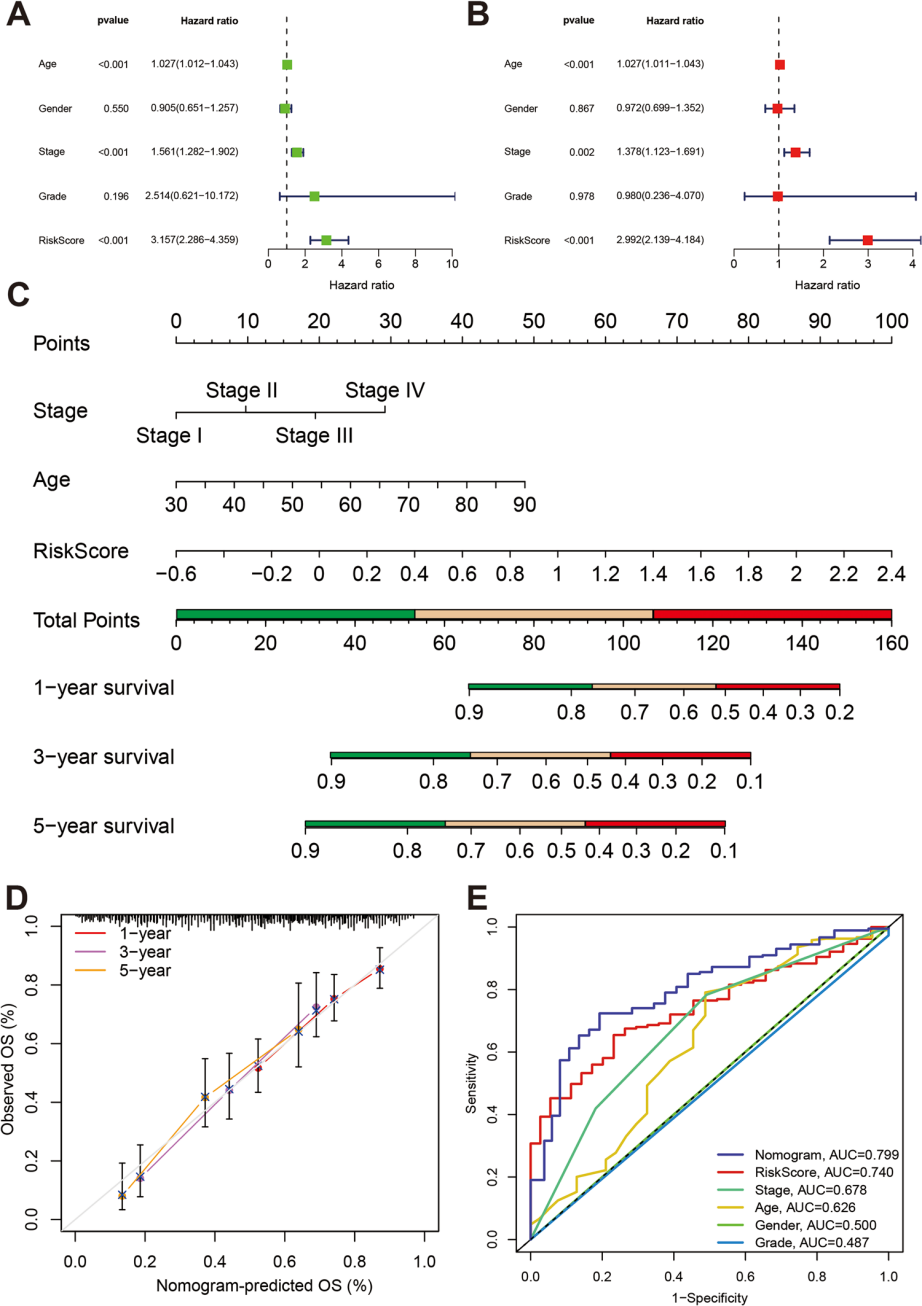
Construction and accuracy testing of nomograms. (**A**) One-way COX regression analysis of risk scores versus clinical factors. (**B**) Multifactor COX regression analysis of risk score versus clinical factors. (**C**) Nomogram constructed with Age, Stage, and RiskScore as risk factors. (**D**) Calibration curves for nomograms. (**E**) ROC curves for nomogram, RiskScore, and other clinical factors.

To construct nomograms for predicting patient survival, we conducted uni-variate and multi-variate Cox regression analyses involving risk scores and clinical factors. Uni-variate Cox regression analyses revealed significant associations between RiskScore (*p* < 0.001, risk ratio [HR] = 3.157, 95% confidence interval [CI] = 2.286–4.359), Clinical stage (*p* < 0.001, HR = 1.561, 95% CI = 1.282–1.902), and Age (*p* < 0.001, HR = 1.027, 95% CI = 1.012–1.043) with Overall Survival (OS) in the TCGA-BLCA cohort (Fig. 4A). In multi-variate Cox regression analyses, RiskScore, Clinical stage, and Age were similarly statistically significant (Fig. 4B).

Nomograms, integrating multiple risk factors, were developed for predicting survival in the TCGA-BLCA cohort. The model incorporated three independent risk factors: age, stage, and RiskScore, and could predict survival probabilities by calculating the cumulative total score for each independent factor for each patient (Fig. 4C). The nomogram's predictive performance was validated through calibration curves and ROC curves, indicating that the actual OS aligned well with the OS predicted by the nomogram at 1, 3, and 5 years. The area under the ROC curve value reached 0.799, demonstrating good predictive performance (Fig. 4D,E, Supplementary Fig. S1C).

### MAPK-related gene prognostic models concerning tumor mutation load and immunotherapy response

**Figure 5:**
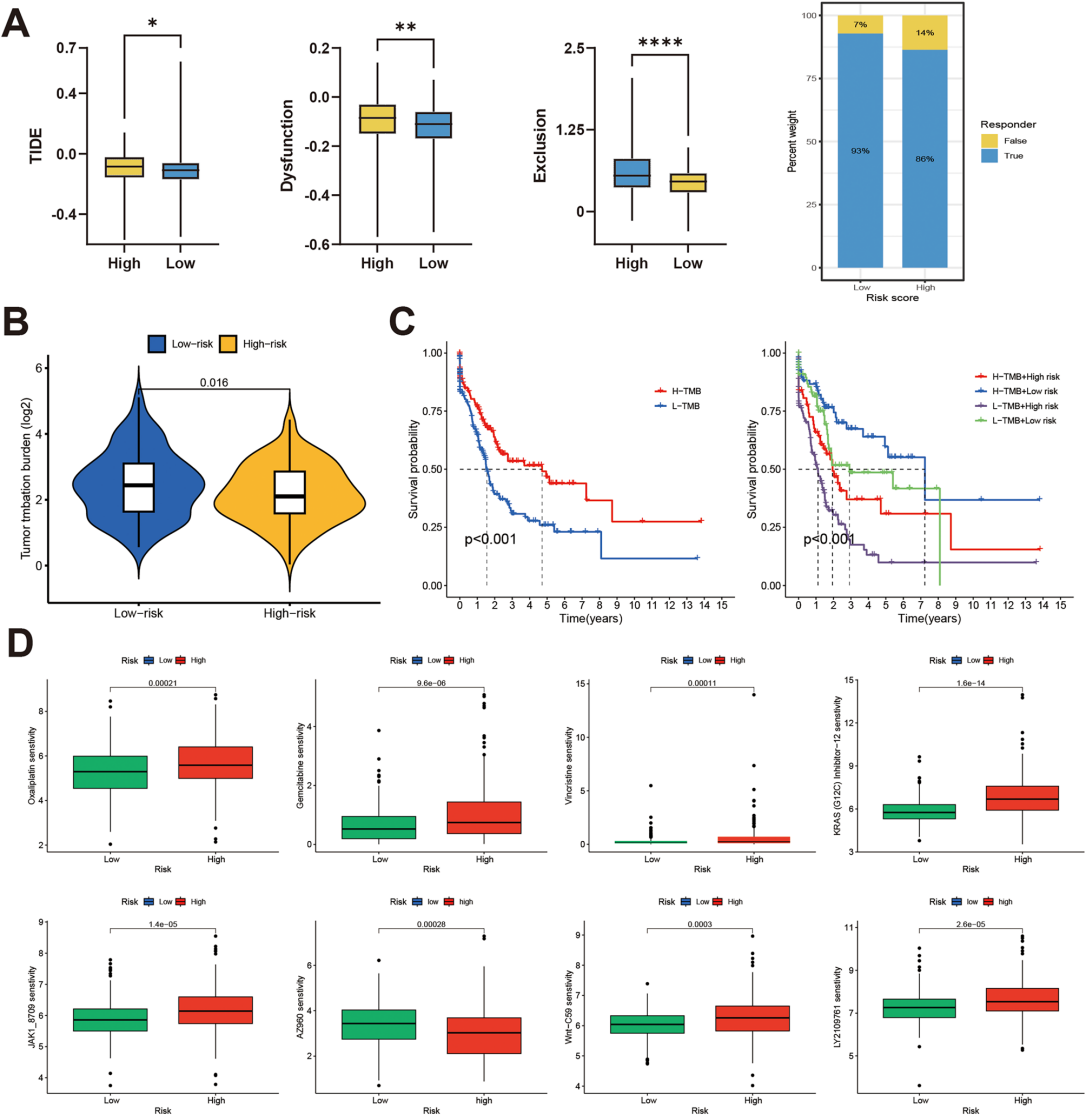
TIDE analysis and mutation assessment. (**A**) TIDE analysis of immune checkpoint inhibitor responses in two subgroups. (**B**) TMB differences between two groups. (**C**) Kaplan–Meier curves between different TMB subgroups. (**D**) Sensitivity analysis of anticancer medications in two groups.

Afterward, we performed a study to compare the variances in immunotherapy reactions among the two subgroups. The findings from Tumor Immune Dysfunction and Exclusion (TIDE) indicated that samples categorized as the low-risk category showed a greater rate of response to immunosuppressive medications (Fig. 5A). Furthermore, we examined the tumor somatic mutation landscapes in two groups. In both subgroups, the results indicated that the mutation rates of TP53, TTN, KMT2D, MUC16, and ARID1A genes were higher than 20%, as shown in Supplementary Fig. S1D. Analysis of TMB status between the two groups showed that TMB was significantly increased in the low-risk group (Fig. 5B). Kaplan–Meier survival analysis showed that the high TMB group had a better prognosis. Notably, patients with low TMB and concomitant high risk had the worst prognosis (Fig. 5C, *p* < 0.001). Ultimately, utilizing the 'oncoPredict' R package, we conducted a comparison of the variances in medication responsiveness among the two cohorts. The findings indicated that the half inhibitory concentrations (IC_50_) values of oxaliplatin, gemcitabine, and vincristine were considerably lower in the low-risk group. This implies that patients with lower risk scores could potentially gain greater advantages from utilizing these medications (Fig. 5D). Among several recognized cancer-related pathway drugs, low-risk scoring cases had significantly lower IC50s for KRAS (G12 C) inhibitor-12, JAK1_8709, Wnt-C59, and LY2109761, and higher sensitivity to AZ960 (Fig. 5D).

### SNV, CNV, and drug sensitivity analysis

**Figure 6:**
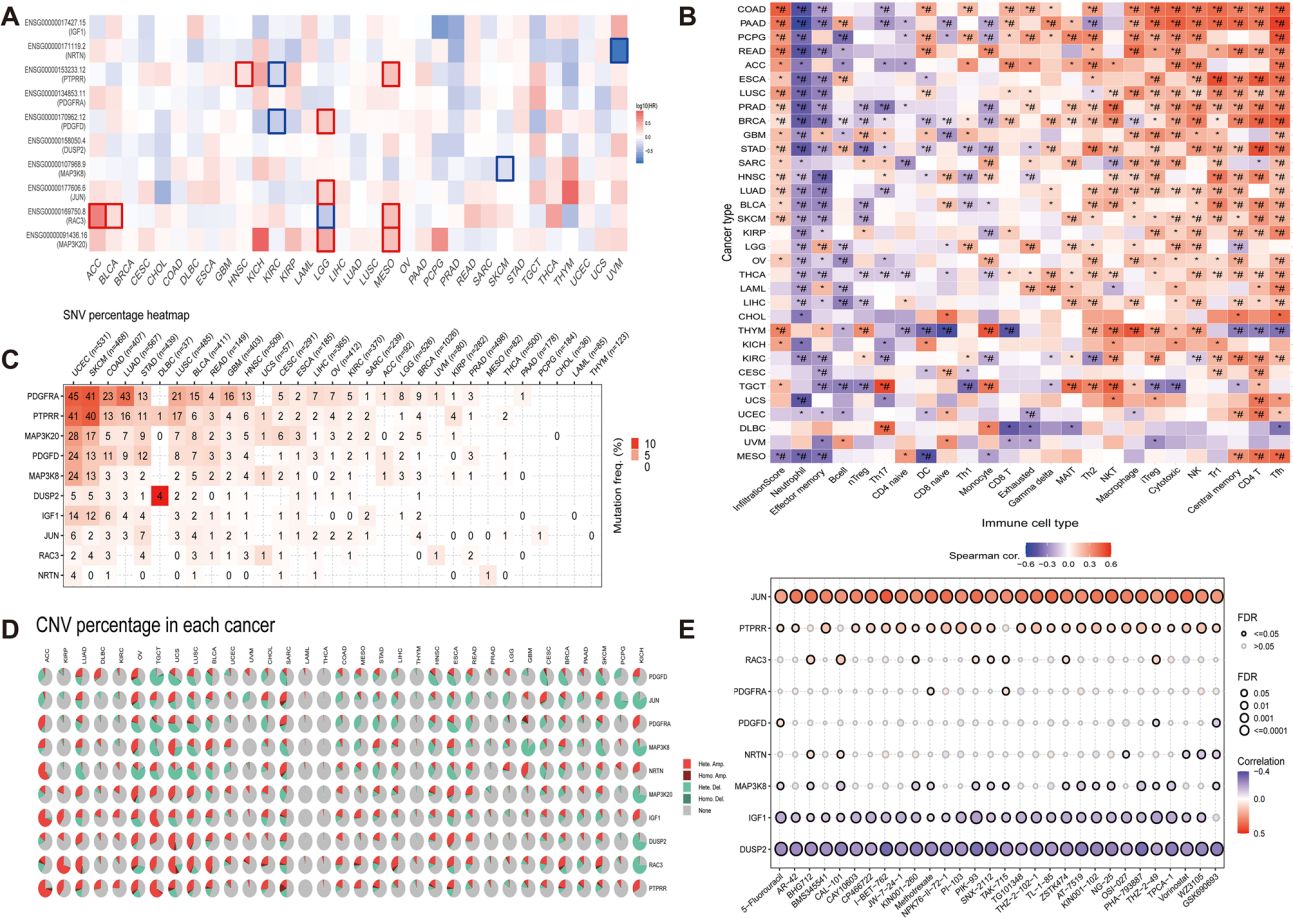
Pan-cancer analysis of model genes was performed using the GEPIA and GSCA websites. (**A**) Heatmap of overall survival analysis of model genes in pan-cancer. (**B**) GSVA analysis of the level of immune cell infiltration in different tumors, with a positive correlation in red and a negative correlation in blue. (**C**) The pie chart illustrates the distribution of SNV of model genes across various tumor types. (**D**) The heatmap provides an overview of the CNV proportions in the model genes across different cancers. It uses a color scheme to indicate various types of CNVs: light red for heterozygous amplification (Hete. amp), dark red for homozygous amplification (Homo. amp), light green for heterozygous deletion (Hete. del), dark green for homozygous deletion (Homo. del), and grey to denote the absence of CNVs. (**E**) Model gene correlation analysis with anticancer drug sensitivity.

We utilized the GEPIA2 website to generate a heat map illustrating the survival analysis for the 10 model genes across 33 different tumors (Fig. 6A). We further investigated the association of 10 model genes with immune cell infiltration in pan-cancer (Fig. 6B). The analysis revealed high expression of Th2 cells, natural killer T cells, macrophage cells, iTreg cells, cytotoxic cells, NK cells, Tr1 cells, central memory cells, CD4 T cells, and Tfh cells in most tumors, indicating a potential association with tumor progression. In contrast, neutrophil and effector memory cells showed low expression. Additionally, SNV and CNV percentage heatmaps for the 10 model genes in 32 tumors were plotted using the GSCA website (Fig. 6C,D). The analysis of heatmaps revealed that PDGFRA and PTPRR demonstrated high SNV across various cancers, whereas PTPRR and RAC3 exhibited elevated CNV in the majority of cancer types. We also examined the association between the 10 model genes and drug sensitivity (Fig. 6E). The analysis revealed that elevated expression of JUN and PTPRR genes was associated with increased drug sensitivity, while IGF1 and DUSP2 genes displayed a negative correlation.

### Expression and clinical relevance of model genes

**Figure 7:**
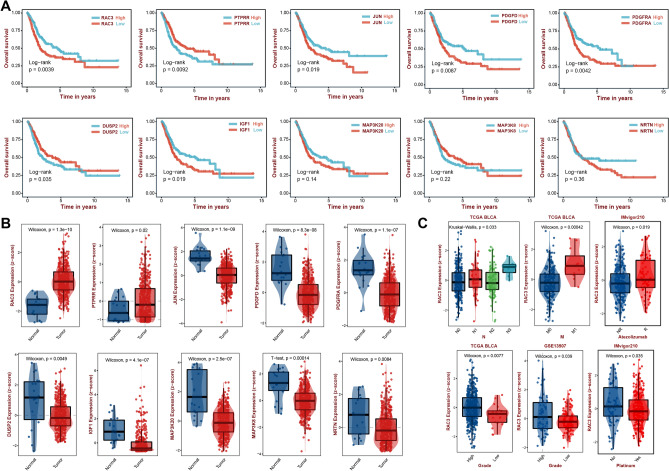
Expression and clinical correlation of model genes. (**A**) Kaplan–Meier curves of 10 model genes. (**B**) Box line plots of the expression of 10 model genes in tumor tissues and normal tissues. (**C**) Correlation of RAC3 with clinical traits.

To further clarify the role of the model genes in the MAPK model, we grouped the samples using these 10 model genes, and subsequently performed Kaplan–Meier survival analysis on the different expression subgroups of the genes (Fig. 7A), which showed that the survival rate was lower in the high expression group of the genes, such as RAC3, JUN, PDGFD, PDGFRA, and IGF1 (*p* < 0.05), while the survival rate was worse in the group with low expression of PTPRR and DUSP2 (*p* < 0.05). Differential expression analysis of 10 genes in tumor and normal tissues showed that RAC3 and PTPRR were highly expressed in tumor tissues, while the rest of the genes were lowly expressed in tumors (Fig. 7B, *p* < 0.05). Interestingly, we found that RAC3 gene was highly expressed in tumors and patients with high RAC3 expression had worse prognosis, which attracted us to analyze it further, and the results showed that RAC3 was highly expressed in patients with lymph node metastasis N3 stage, distant metastasis M1 stage, and high grade of pathology (Fig. 7C, *p* < 0.05), which may suggest that RAC3 plays a role in the metastatic process of BLCA, or its expression may be associated with higher tumor aggressiveness in BLCA.IMvigor210 cohort analysis found higher RAC3 expression in Atezolizumab-responsive patients and platinum non-responsive patients (Fig. 7C, *p* < 0.05), which suggests that RAC3 expression may be a potential predictor of response to immunotherapy and chemotherapy, which deserves to be further explored, and can be further explored by future studies the specific role of RAC3 in BLCA and its potential application in disease prognosis and treatment personalization.

### GSEA analysis of model genes in BLCA

**Figure 8:**
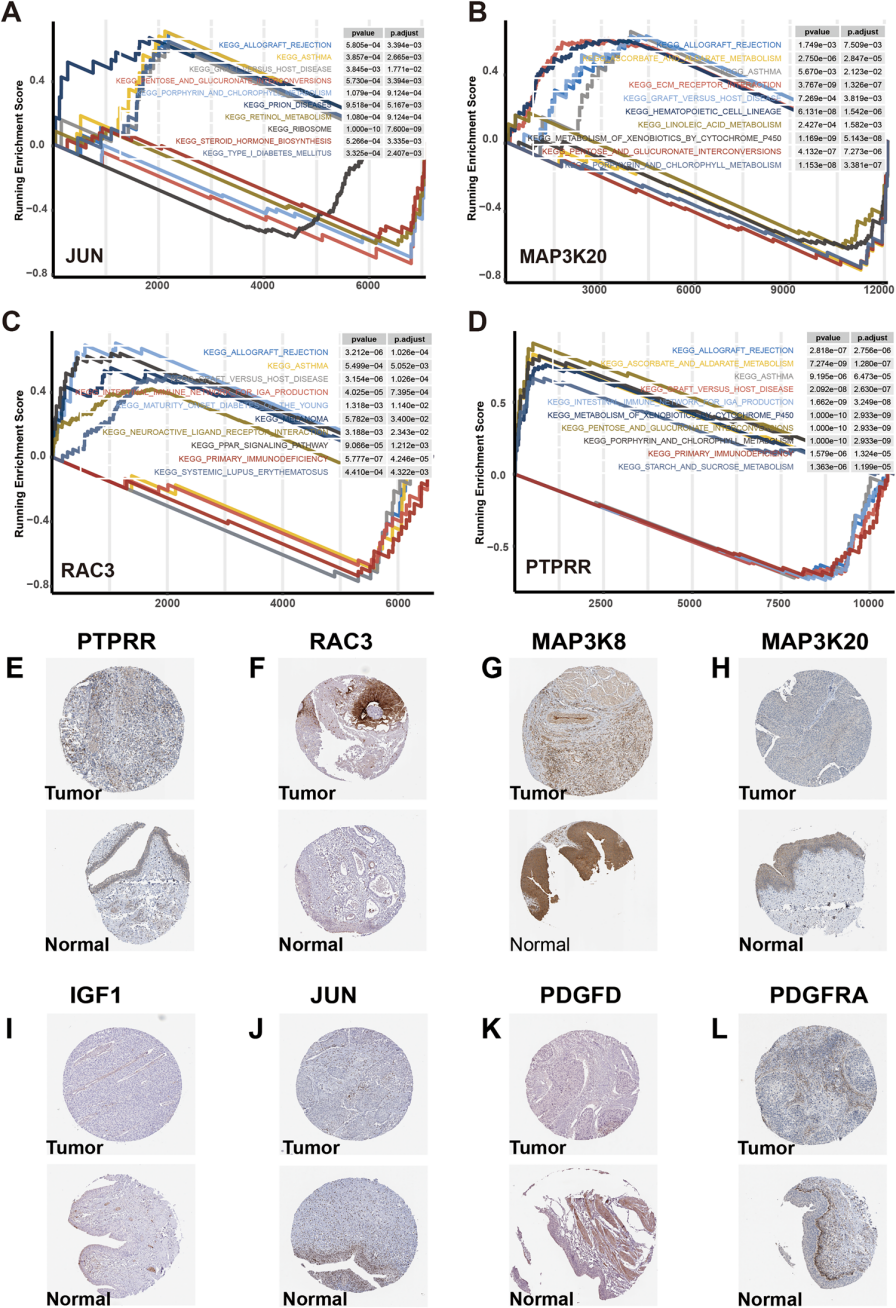
Immunohistochemical images of model genes from the HPA database. The GSEA result of (**A**) JUN, (**B**) MAP3K20, (**C**) RAC3, and (**D**) PTPRR. Immunohistochemical images of (**E**) PTPRR. (**F**) RAC3. (**G**) MAP3K8. (**H**) MAP3K20. (**I**) IGF1. (**J**) JUN. (**K**) PDGFD. (**L**) PDGFRA.

Significant enrichments in different pathways were revealed through the analysis of GSEA conducted on the two groups. In Supplementary Fig. S2A, the group at high risk showed notable enhancement in pathways associated with Cytokine-cytokine receptor interaction, ECM receptor interaction, focal adhesion, Neuroactive ligand-receptor interaction and receptors, and Regulation of actin cytoskeleton. The group with low risk exhibited notable enhancement in pathways linked to Linoleic acid metabolism, Oxidative phosphorylation, Pentose and glucuronate interconversions, and Ribosome (Supplementary Fig. S2B). Further analysis of the model gene GSEA-KEGG pathway revealed enrichment of each gene in numerous pathways. The top 5 elevated and reduced pathways for each gene were selected for presentation (Supplementary Fig. S2C–H and Fig. 8A–D). Single-gene GSEA revealed that the model genes play roles in different tumor-associated pathways. And interestingly these 10 model genes were all enriched in pathways such as metabolism and immune system, which may indicate that these genes play important roles in regulating tumor-associated metabolic processes, and immune system activities. The discovery of these pathway enrichments provides new clues for understanding tumor biology and may reveal the potential of these genes as therapeutic targets.

### Immunohistochemical images of model genes from the HPA database

For the validation of the protein expression of the 10 prognostic genes, we retrieved the expression data of the model genes from the HPA database. Compared to normal tissues, tumor tissues displayed a significant increase in staining intensity for *PTPRR* and *RAC3* (Fig. 8E,F). Conversely, genes such as *MAP3K8*, *MAP3K20*, *IGF1*, *JUN*, *PDGFD*, and *PDGFRA* displayed low staining intensity relative to normal tissues (Fig. 8G–L).

### qRT-PCR-based validation of differential mRNA expression in BLCA clinical samples

**Figure 9:**
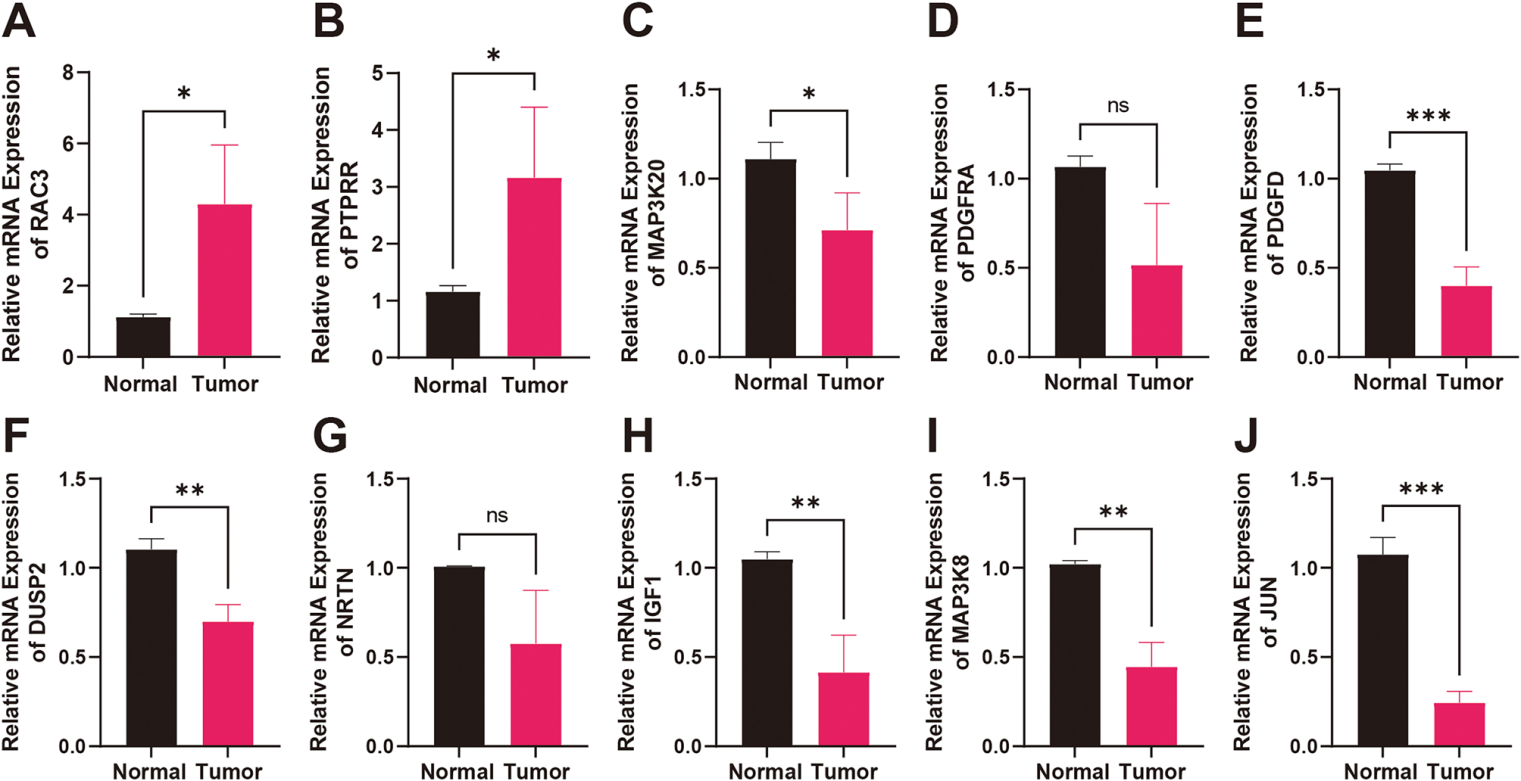
qRT-PCR results of clinically collected tissues. (**A**) RAC3. (**B**) PTPRR. (**C**) MAP3K20. (**D**) PDGFRA. (**E**) PDGFD. (**F**) DUSP2. (**G**) NRTN. (**H**) IGF1. (**I**) MAP3K8. (**J**) JUN.

To further validate the expression of the model genes in clinical samples, we gathered 10 pairs of BLCA and paracancerous tissues. Subsequently, we extracted RNA from these samples, performed reverse transcription, and conducted qRT-PCR. The results demonstrated that the genes *RAC3* and *PTPRR* were significantly overexpressed compared to normal tissues (Fig. 9A,B). In contrast, genes such as *MAP3K20*, *PDGFD*, *DUSP2*, *IGF1*, *MAP3K8*, and *JUN* exhibited significant downregulation relative to normal tissues. However, *PDGFRA* and *NRTN* did not show statistically significant differences (Fig. 9C–G).

## Discussion

Metastasis-prone MIBC continues to present a significant clinical challenge due to its high mortality rate, with the current first-line treatment typically involving platinum-based chemotherapy. However, the overall prognosis remains poor^34^. Despite the increasing use of inhibitors targeting immune checkpoints such as PD-1 and PD-L1 in the treatment of BLCA, the efficacy of immunotherapy is still suboptimal, with a remission rate of about 25%^35^. Advances in next-generation sequencing technologies over the past few years have underscored the importance of identifying novel molecular biomarkers in BLCA for accurately predicting patient prognosis, a critical aspect of clinical decision-making^36^. Newly discovered evidence indicates that the MAPK pathway is a viable option for treating cancer, while the ERK pathway stands out as a significant and widely employed area of interest in clinical practice^37^. Additionally, the JNK and p38 pathways, while playing crucial regulatory roles, present challenges in predicting cancer cell responses to targeted therapies and chemotherapy due to their dependence on upstream and downstream environments^38^.

In this investigation, we identified 26 prognostic genes associated with BLCA survival through COX analysis within the MAPK pathway-related genes. Subsequently, a novel prognostic signature for bladder cancer patients was developed utilizing LASSO-COX analysis, focusing on 10 MAPK pathway-related prognostic genes (*NRTN, RAC3, JUN, IGF1, DUSP2, MAP3K8, PDGFD, MAP3K20, PTPRR,* and *PDGFRA*). The validation of this signature was confirmed both internally and externally, indicating that patients with high-risk scores had notably worse overall survival. The prognostic model demonstrated strong performance with a higher AUC. Both uni-variate and multi-variate Cox regression analyses confirmed that the correlation model of MAPK served as a separate predictor for the overall survival in BLCA. To improve clinical applicability, nomograms were constructed, and calibration curves showed well-validated and stable predictive performance. To explore potential molecular mechanisms, we further performed tumor immune landscape and mutation landscape analysis, clinicopathological information analysis, and GSEA. Finally, we substantiated the expression of model genes in BLCA, further confirming the differential expression of MAPK pathway-related genes.

In this study, we reveal an important link between tumor immune profiles and patient prognosis through an in-depth analysis of the differences in immune microenvironment characteristics and response to immunotherapy between the two-risk scoring groups. The high-risk scoring group exhibited higher StromalScore, ImmuneScore, and ESTIMATEScore, as well as an increase in specific immune cell subtypes, suggesting a more complex and immunosuppressive tumor microenvironment. Furthermore, we found that patients in the high-risk group had a lower response rate to immunosuppressive drugs, but increased TMB was associated with a better prognosis in patients in the low-risk group. These findings highlight the central role of the tumor microenvironment in tumor progression and immunotherapy outcomes and point to the potential of using tumor microenvironment characteristics for patient risk stratification and treatment selection. Higher risk scores were associated with a more complex tumor microenvironment and immunosuppressive status, whereas an increase in TMB was observed in the low-risk scoring group, suggesting that TMB could serve as a useful biomarker to assist in risk scoring to optimize treatment decision-making. The combination of risk scores, immune profiles and TMB assessment results based on gene expression data from patient tumor samples in daily practice can help determine a patient's suitability for immunotherapy. In clinical management, the model can be used to select the most appropriate immunotherapy strategy, prognostic assessment and optimize drug selection.

Prognostic differences between different risk subgroups are often driven by underlying molecular mechanisms and pathways^39,40^. Our GSEA results showed that the group at high risk showed notable enhancement in pathways associated with Cytokine-cytokine receptor interaction, ECM receptor interaction, focal adhesion, Neuroactive ligand-receptor interaction and receptors, and Regulation of actin cytoskeleton. The previous study showed that Cytokine-cytokine receptor interaction regulates immune response and inflammation by activating various signaling pathways, including the MAPK pathway, cytokines such as IL-6 and TNF-α activate the MAPK pathway, leading to increased inflammatory response, which plays an important role in the development of autoimmune diseases and cancers, and that enrichment of this pathway in high-risk groups may imply a stronger inflammatory state or aberrant immune activation^41,42^. ECM receptor interaction and Focal adhesion both are associated with extracellular matrix (ECM) and cell adhesion, and previous reports have shown that MAPK signaling is associated with ECM production and degradation, thereby promoting cell migration and proliferation^43–46^, which may be a reflection of the worse prognosis of high-risk groups. Cytoskeletal regulation is critical for cell shape, motility, and division. The MAPK pathway affects cell migration by regulating the activity of proteins associated with cytoskeletal reorganization and invasiveness^47,48^, which is particularly important in tumor spread and metastasis.

Among the 10 model genes, *RAC3* stands out as one of the three isoforms within the Rho GTPase subfamily^49,50^. Studies have demonstrated that *RAC3* enhances tumor cell proliferation, migration, and invasion by activating the JAK/STAT signaling pathway^51,52^. Furthermore, high expression of RAC3 predicts a poor prognosis of BLCA^53^. Our study also confirmed high *RAC3* expression, which provides a basis for using *RAC3* as a therapeutic target for BLCA. Conversely, *PTPRR* exhibits a dual function. Suppression of *PTPRR* expression in rectal cancer triggers the Ras/ERK/c-Fos signaling pathway, thereby facilitating the development of rectal carcinogenesis^54^. In ovarian cancer, PTPRR functions as a suppressor of tumors by dephosphorylating and rendering β-conjugated proteins inactive^55^, suggesting a potentially protective role. *NRTN* is a ligand of the neurotrophic factor family, and recent studies have shown that NRTN is associated with rectal, pancreatic, and hepatocellular cancer progression^56–58^. *IGF-1* is a growth hormone target gene that binds to and activates the receptor tyrosine kinase IGF1 receptor (IGF1R)^59^. Multiple studies have demonstrated its association with resistance to anticancer drugs and highlighted the potential benefits of targeting IGF1 in anticancer therapy^60,61^. Additionally, a large case–control study has indicated a significant association between IGF1 and a reduced risk of BLCA^62^. *MAP3K8* is an oncogene encoding a member of the serine/threonine protein kinase family, and studies have shown that high levels of MAP3K8 phosphorylation are associated with progression and poor prognosis in patients with BLCA^63^. Studies have indicated that *PDGFD*, a part of the platelet-derived growth factor family, is linked to gemcitabine resistance in BLCA patients and unfavorable prognosis in advanced uroepithelial carcinoma and pancreatic ductal adenocarcinoma (PDAC)^64,65^. *PDGFRA*, a receptor for tyrosine kinase, is frequently mutated in gastrointestinal mesenchymal stromal tumors (GIST) and serves as a target for anticancer medications like avastinib^66,67^. *JUN* has been implicated in APF-mediated growth inhibition of bladder tumor cells and is a potential target of APF in patients with invasive BLCA^68^, and overexpression of *JUN* protein is also closely associated with the invasive growth of BLCA^69^. *DUSP2* belongs to the nuclear DUSP family, specifically type I, and it inhibits the activation of MAPK while having a crucial function in immune processes, inflammatory responses, and the advancement of cancer. Deletion of *DUSP2* connotes a poor prognosis for patients with BLCA^70^. *MAP3K20*, a member of the MAP3K subfamily^71,72^, has been associated with the regulation of HCC cell proliferation and apoptosis^73^. Finally, high *MAP3K20* expression has been found to promote cancer progression in gastric, breast, bladder, and colorectal cancers^74–77^.

Taken together, we have developed and validated a novel prognostic model centered on MAPK pathway-associated genes. Despite its strengths, the study has several limitations. Initially, it was a retrospective analysis using TCGA and GEO databases, necessitating additional prospective real-world data to confirm the accuracy of the MAPK-related gene model. Furthermore, our validation was limited to basic preliminary qPCR analysis, requiring further experiments related to biological functions to elucidate the roles and mechanisms of these genes in BLCA development. Lastly, although the model was validated using the GEO database, further extensive integration tests with centralized cohorts are essential to comprehensively evaluate the model's performance.

## Conclusion

In conclusion, we identified MAPK pathway-associated DEGs by comprehensive analysis of the TCGA-BLCA dataset and revealed 10 genes significantly associated with the prognosis of BLCA patients by survival analysis. By building predictive models based on these genes, we successfully distinguished subgroups of BLCA patients with different survival expectations. In addition, our analysis highlighted the close association of these prognostic genes with the tumor microenvironment and immune response, providing potential biomarkers for future targeted therapy and immunotherapy. We believe that these findings provide new insights into the molecular mechanisms of BLCA research and clinical management.

### Supplementary Information

Supplementary Figure S1.

Supplementary Figure S2.

Supplementary Table S1.

Supplementary Table S2.

Supplementary Table S3.

## Data Availability

Public data used in this work can be acquired from the TCGA Research Network portal (https://portal.gdc.cancer.gov/), Gene Expression Omnibus (http://www.ncbi.nlm.nih.gov/geo/), Kyoto Encyclopedia of Genes and Genomes (https://www.genome.jp/kegg/pathway.html/), Genomics of Drug Sensitivity in Cancer (https://www.cancerrxgene.org/), Gene Expression Profiling Interactive Analysis (http://gepia.cancer-pku.cn/), Gene Set Cancer Analysis (https://guolab.wchscu.cn/GSCA/), and The Human Protein Atlas database (http://www.proteinatlas.org/).
