# Supplementary material for: Predicting bladder cancer survival with high accuracy: insights from MAPK pathway-related genes
Source: Sci Rep. 2024 May 7;14:10482. doi: 10.1038/s41598-024-61302-0 (PMC11076554; doi:10.1038/s41598-024-61302-0)
Supplement: Supplementary file 1 — Supplementary Figure S1. [file 41598_2024_61302_MOESM1_ESM.pdf]

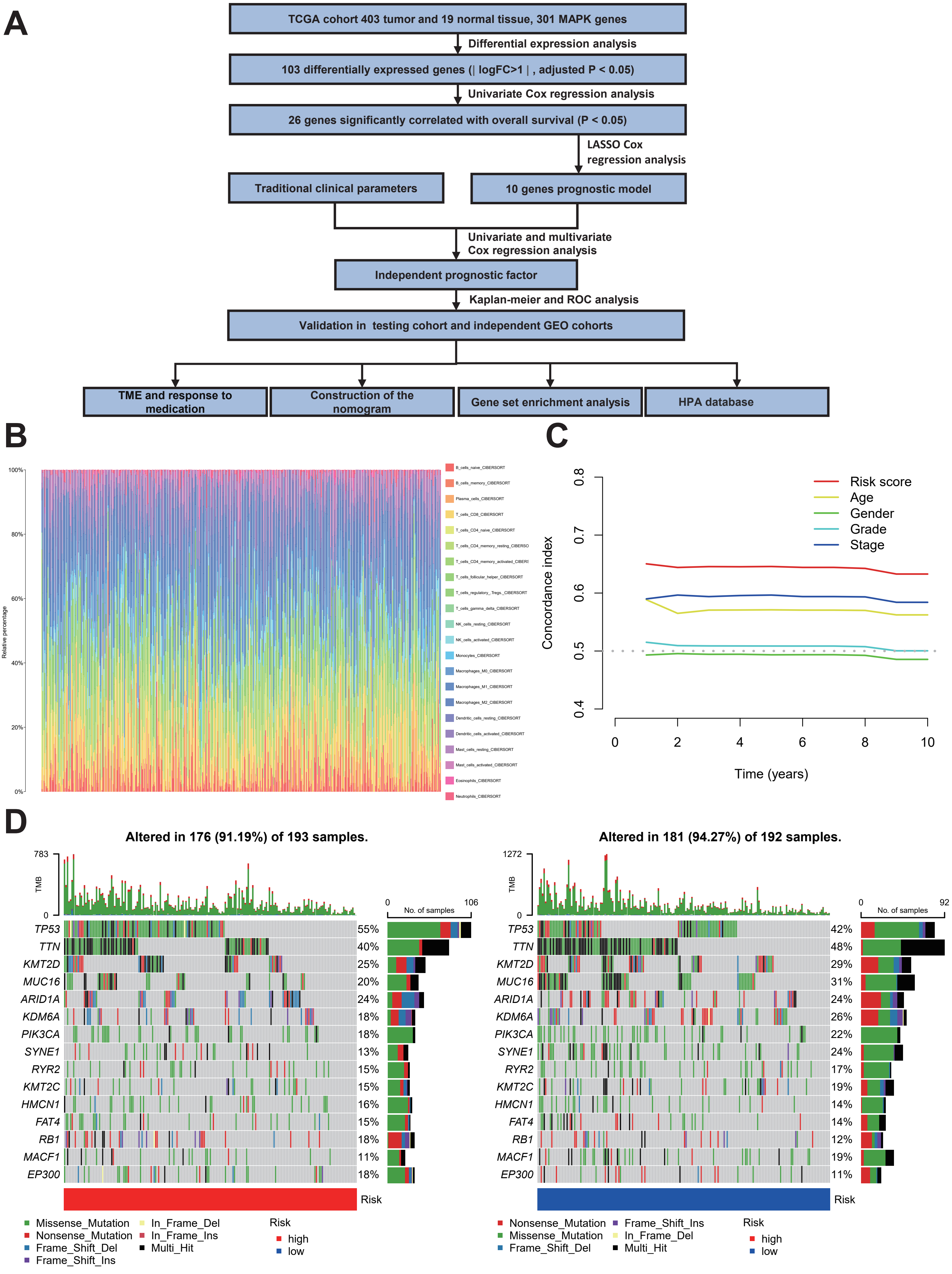

Supplementary figures1. (A)The workflows of this study.(B)TCGA-BLCA immune cell infiltration landscape. (C)C-index values for prognostic factors.(D)Landscape of somatic mutations in high-risk and low-risk groups.
