# Supplementary material for: Predicting bladder cancer survival with high accuracy: insights from MAPK pathway-related genes
Source: Sci Rep. 2024 May 7;14:10482. doi: 10.1038/s41598-024-61302-0 (PMC11076554; doi:10.1038/s41598-024-61302-0)
Supplement: Supplementary file 2 — Supplementary Figure S2. [file 41598_2024_61302_MOESM2_ESM.pdf]

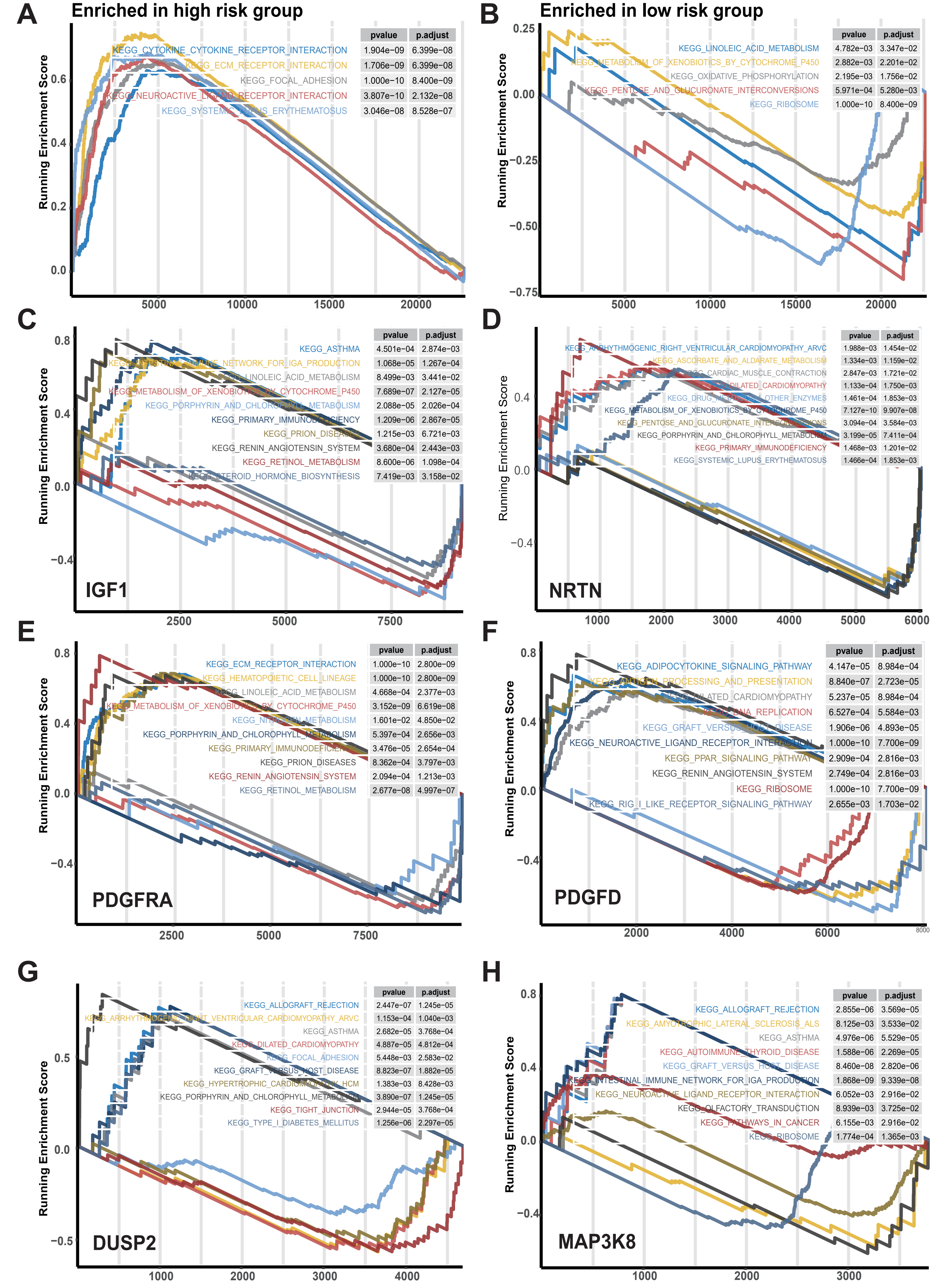

Supplementary Figures S2 . GSEA analysis of modeled genes in the BLCA. The GSEA result of (A) High-risk group. (B) Low-risk group. (C) IGF1. (D) NRTN. (E) PDGFRA. (F) PDGFD. (G) DUSP2. (H) MAP3K8.
